# Eine klassische Diagnose mit unerwartetem Hintergrund

**DOI:** 10.1007/s00104-026-02455-8

**Published:** 2026-02-09

**Authors:** Laurent Heesen, Erik Ranschaert, Muriel Burlet, Eric Lemaire, Maud Collin, Susanna Kreitz, Marike Leijs

**Affiliations:** 1 Klinik für Dermatologie und Allergologie, Medizinische Fakultät, RWTH Aachen, Aachen, Deutschland; 2 Klinik für Radiologie, St. Nikolaus Hospital Eupen, Eupen, Belgien; 3 Universität Gent, Gent, Belgien; 4 Klinik für Pathologie, St. Nikolaus Hospital Eupen, Eupen, Belgien; 5 Klinik für Chirurgie, St. Nikolaus Hospital Eupen, Eupen, Belgien; 6 Klinik für Pädiatrie, St. Nikolaus Hospital Eupen, Eupen, Belgien; 7 Klinik für Dermatologie, St. Nikolaus Hospital Eupen, Eupen, Belgien; 8 Klinik für Dermatologie, Maastricht University Medical Centre+(MUMC+), P. Debyelaan 25, 6229 HX Maastricht, Niederlande

## Anamnese und klinischer Befund

Eine 13-jährige Patientin stellte sich mit seit 2 Wochen zunehmenden, diffusen abdominellen Schmerzen vor, anfänglich mit leichter Betonung im rechten Unterbauch. Seit 5 Tagen bestanden subfebrile Temperaturen (37,5–38,3 °C), zudem seit 4 Wochen Fatigue und Appetitlosigkeit. Stuhlgang weitgehend unauffällig. Keine Medikamenteneinnahme oder Auslandsreisen, Anamnese und Familienanamnese unauffällig bis auf eine Mykoplasmeninfektion vor 6 Wochen. Klinisch zeigte sich ein weicher, diffus druckdolenter Bauch mit maximalem Schmerz im rechten Unterbauch, lokaler Abwehrspannung sowie positivem McBurney‑, Lanz‑, Blumberg- und deutlich positivem Rovsing-Zeichen.

Die Laboruntersuchungen ergaben keine pathologischen Auffälligkeiten:

Das C‑reaktive Protein (CRP), die Blutsenkungsgeschwindigkeit (BSG) sowie die Leukozytenzahl und -differenzierung lagen im Normbereich.

Auch die Leber- und Nierenfunktionsparameter sowie die Laktatdehydrogenase (LDH) zeigten keine Auffälligkeiten.

Die Körpertemperatur war mit 37,8 °C leicht erhöht.

Das Urinsediment zeigte keine pathologischen Veränderungen.

**Abb. 1:**
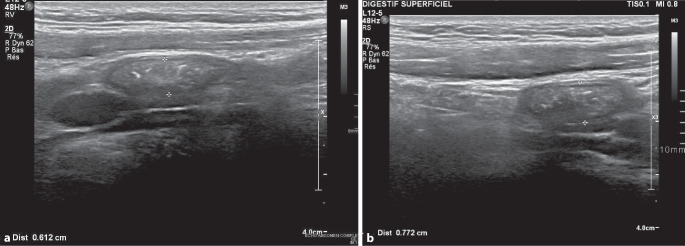
**a** Der Ultraschall zeigt ein Längsbild des Blinddarms mit einer linearen Hochfrequenzsonde (L12‑5, 48 Hz). Der Blinddarm war komplett sichtbar und hatte distal eine Breite von etwa 6,1 mm (normaler Grenzwert ≤ 6 mm). Die Wandschichten waren erhalten. Die Kompressibilität war deutlich vermindert. Im Lumen wurde stark echogenes Material mit einer unregelmäßigen röhrenförmigen Struktur gefunden. Es gab keine freie Flüssigkeit im rechten Unterbauch, keine vergrößerten Lymphknoten und keine auffällige entzündliche Infiltration des umgebenden peritonealen Fettgewebes. Bei gezielter Sonopalpation war eine leichte Druckschmerzhaftigkeit spürbar. **b** Die Ultraschalluntersuchung am nächsten Tag bestätigte erneut die früheren Befunde, und eine Dicke von 7,7 mm wurde gemessen. Der Blinddarm war nicht komprimierbar. Erneut waren einige interne unregelmäßige Reflexionen zu sehen

Eine Sonographie des Abdomens (Abb. 1a) wurde durchgeführt.

Unter Verwendung eines hochfrequenten linearen Schallkopfs (L12‑5, 48 Hz) konnte der Appendix in Längsrichtung vollständig dargestellt werden.

Distal betrug der maximale Durchmesser etwa 6,1 mm.

Im Lumen zeigte sich stark echogenes Material mit irregulärer, tubulärer Struktur.

Die Sonographie der übrigen Abdominalorgane ergab keine pathologischen Befunde.

Die Patientin wurde bei unauffälligen Entzündungsparametern und auf eigenen Wunsch noch am selben Tag entlassen.

## Weiteres Prozedere

Am Folgetag erfolgte eine erneute klinische Beurteilung, im Rahmen derer die Patientin aufgrund progredienter abdomineller Beschwerden stationär aufgenommen wurde. Bei zunehmenden, rechtsseitig betonten Unterbauchschmerzen und einer sonographisch nachgewiesenen Kaliberzunahme der Appendix (Abb. 1b) mit Verdacht auf eine luminale Obstruktion durch einen Appendikolithen wurde eine diagnostisch-therapeutische Laparoskopie durchgeführt.

Intraoperativ zeigte sich ein leicht ödematöser Appendix mit vermehrter Gefäßzeichnung, vereinbar mit einer Appendizitis. Hinweise auf eine perforierte Appendizitis bestanden nicht. Die endoskopische Appendektomie erfolgte mittels Stapler. Die übrigen abdominellen Organe einschließlich Ovarien, Adnexe und terminales Ileum zeigten sich unauffällig.

## Wie lautet Ihre Diagnose?

## Definition/Diskussion

Gastrointestinale Parasiten können eine Ursache der Appendizitis sein, auch wenn sie deutlich seltener auftreten als eine luminale Obstruktion durch Appendikolithen, die etwa 40 % der Fälle ausmacht [1, 2]. Parasiten können auch das klinische Bild einer Appendizitis imitieren; postoperativ findet sich dann ein histologisch gesunder Appendix (negative Appendektomie) [3].

**Diagnose:** reaktive Appendikopathie bei *Enterobius-vermicularis*-Infestation

Zu den häufigsten Wurmerkrankungen in Ländern mit gemäßigtem Klima zählt *Enterobius vermiculari*s (*E. vermicularis*) mit weltweit über eine Milliarde betroffenen Menschen [4, 5]. Der Zusammenhang zwischen einer *Enterobius*-Infektion und der Entstehung einer Appendizitis bzw. das Vorliegen eines Zufallsbefundes eines Wurmbefalls bei einer Appendizitis anderer Genese bleibt jedoch umstritten [2]. Patienten mit einem Befall des Appendix durch *E.vermicularis * können sich mit klinischen Symptomen einer Appendizitis präsentieren. Bei einer negativen Appendektomie wie in unserem Fall wird dabei wahrscheinlich eine sog. appendikuläre Kolik hervorgerufen [5].

**Abb. 2:**
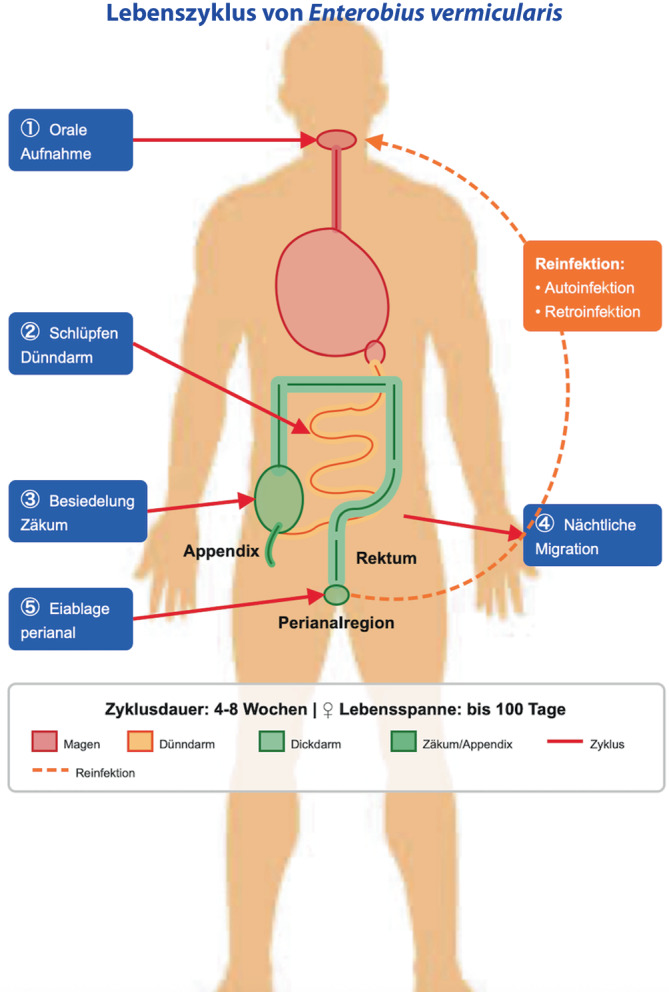
Lebenszyklus von Enterobius vermicularis (*E.* *vermicularis)*. Dieser läuft wie folgt ab: ① Orale Aufnahme: Infektiöse Eier werden über kontaminierte Hände, Bettwäsche oder andere Gegenstände oral aufgenommen. ② Schlüpfen im Dünndarm: Im Duodenum schlüpfen die Larven unter Einwirkung der Verdauungsenzyme aus den Eiern. ③ Besiedelung des Zäkums: Die Larven wandern in das Zäkum, wo sie heranreifen und sich paaren. ④ Nächtliche Migration: Gravide Weibchen migrieren nachts durch den Dickdarm in die Perianalregion. ⑤ Perianale Eiablage: Die Weibchen deponieren ihre Eier in den Perianalfalten und verursachen durch ihre Migration einen ausgeprägten Pruritus ani. Reinfektion: Der Pruritus begünstigt eine Autoinfektion. Neu geschlüpfte Larven können zudem von der perianalen Haut in das Rektum zurückwandern. (Diese Grafik wurde mithilfe von Claude [Anthropic Public Benefit Corporation, San Francisco, CA, USA] einem Sprachmodell der künstlichen Intelligenz erstellt. Die Informationen über den Lebenszyklus stammen aus [6])

*E. vermicularis* zählt zu den Parasiten, deren gesamter Lebenszyklus im Menschen stattfindet [3]. Nach der oralen Aufnahme embryonierter Eier schlüpfen die Larven im Dünndarm und erreichen anschließend das Kolon; im Bereich von Zäkum und Appendix entwickeln sich die Würmer zur adulten Form und haften an der Mukosa [4]. Die nächtliche Migration der Weibchen in die Perianalregion erfolgt, da die dort herrschenden externen Bedingungen für die Reifung der Eier essenziell sind. Dies führt zu perianalem Pruritus und begünstigt die Autoinfektion [4]. Neu geschlüpfte Larven können zudem von der perianalen Haut in das Rektum zurückwandern ([4]; vgl. Abb. 2). Die Transmission erfolgt somit primär fäkal-oral. Weitere Übertragungswege bestehen durch kontaminierte Kleidung, Bettwäsche und Gegenstände, was die Bedeutung konsequenter Hygienemaßnahmen unterstreicht [4]. Hierzu zählen gründliches Händewaschen, täglicher Wechsel von Unter- und Bettwäsche, die Reinigung der perianalen Region, das Vermeiden von Nägelkauen sowie die ausschließliche Nutzung persönlicher Textilien [4].

*E. vermicularis* ist *eine der *häufigsten Wurmerkrankungen in Ländern mit gemäßigtem Klima und betrifft weltweit über eine Milliarde Menschen [4, 5]. Am häufigsten sind Kinder von *E. vermicularis* betroffen: In einer populationsbasierten Studie aus der Ostslowakei zeigte *E. vermicularis* eine altersabhängige Prävalenz von 0,97 % bei Kindern zwischen 5 Monaten und 2 Jahren, 5,03 % bei 3‑ bis 6‑Jährigen und 3,91 % bei 7‑ bis 15-Jährigen, wobei die höchste Befallsrate im Vorschulalter auftritt und v. a. auf unzureichende Umsetzung der Hygienemaßnahmen zurückzuführen ist [6]. Eine weitere Studie beschreibt den Prävalenzgipfel im Alter von 5 bis 14 Jahren [7]. Kinder über 14 Jahre und Erwachsene sind hingegen viel weniger betroffen [4].

Die Diagnostik der Enterobiasis beruht neben der Anamnese mit intermittierendem perianalem Pruritus auf der Inspektion von Kleidung, Bettwäsche und Analregion, wobei adulte Würmer gelegentlich makroskopisch sichtbar sind [4]. Der Goldstandard zur Diagnostik ist der perianale Scotch-Tape-Test, der morgens vor der Körperhygiene durchgeführt wird, indem ein Klebeband mehrfach auf die analen und perianalen Hautbereiche aufgebracht wird, um dort abgelegte Wurmeier zu erfassen und anschließend durch mikroskopischen Nachweis die Diagnose zu sichern [4]. Die Sensitivität steigt deutlich, wenn Proben an 3 aufeinanderfolgenden Tagen entnommen werden, während Stuhluntersuchungen, serologische Verfahren und Blutbildveränderungen diagnostisch nicht hilfreich sind [4].

Bei Patienten mit Verdacht auf eine luminale Parasitenbesiedlung des Appendix können zusätzlich bildgebende Verfahren zur Diagnosesicherung beitragen. Die Wahl des bildgebenden Verfahrens orientiert sich an der etablierten Empfehlung zur Bildgebung bei Appendizitis. Die Sonographie stellt insbesondere bei pädiatrischen Patienten aufgrund der fehlenden ionisierenden Strahlung ein bevorzugtes diagnostisches Verfahren dar und weist für den Nachweis einer akuten Appendizitis eine Spezifität von 71–94 % sowie eine Sensitivität von 81–98 % auf [1]. Der direkte Nachweis des Parasiten ist sonographisch schwierig; hochechogenes, röhrenförmiges Material im Appendixlumen kann jedoch auf seine Anwesenheit hindeuten. In unklaren Fällen kann eine Magnetresonanztomographie (MRT) zur Diagnosesicherung beitragen [1]. Bisher existieren keine belastbaren Daten, die eine bestimmte Methode als überlegen für den spezifischen Nachweis einer parasitären Besiedlung des Appendix aufweisen.

Die medikamentöse First-line-Therapie besteht in der Einmalgabe von Mebendazol, das gemäß Leitlinie nach 14 und 28 Tagen erneut verabreicht werden sollte, um autoinfektionsbedingt verbliebene Eier sicher zu eliminieren [4]. Bei chronisch rezidivierender Enterobiasis wird empfohlen, alle Haushaltsmitglieder und eventuelle Sexualpartner alle 14 Tage über einen Zeitraum von 16 Wochen mitzubehandeln; dieses Vorgehen sollte idealerweise in Zusammenarbeit mit einem spezialisierten Zentrum erfolgen [4].

## Abschließend therapeutisches Vorgehen

Bei der vorgestellten Patientin erfolgte aufgrund des klinischen und sonographischen Verdachts auf eine Appendizitis eine laparoskopische Appendektomie.

**Abb. 3:**
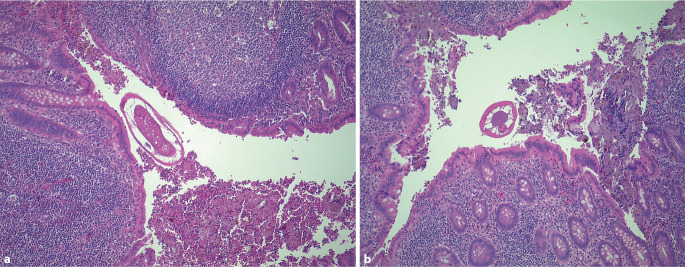
**a,** **b** Lumen des Appendix, das Koprolithen enthält. Die Mukosa weist einige reaktive lymphatische Follikel auf. Die übrigen Wandschichten zeigen keine Auffälligkeiten. Das Lumen enthält *Enterobius vermicularis* (Madenwurm)

Histologisch (Abb. 3a, b) zeigten sich reaktive lymphatische Follikel in der Mukosa bei ansonsten unauffälligen Wandschichten; im Lumen konnte *Enterobius vermicularis* (*E. vermicularis*) nachgewiesen werden.

Postoperativ wurden die Patientin sowie ihre engen Kontaktpersonen mit Mebendazol (100 mg oral, Wiederholung nach 2 Wochen) antiparasitär behandelt.

Die primäre Stuhldiagnostik ergaben keine pathologischen Befunde – Parasiten oder Eier konnten mikroskopisch nicht nachgewiesen werden. Ein perianaler Scotch-Tape-Test wurde retrospektiv nicht durchgeführt.

## Fazit für die Praxis

Der vorliegende Fall unterstreicht, dass bei abdominellen Beschwerden, die eine Appendizitis imitieren, im Kindes- und Jugendalter auch seltene Ursachen – wie ein parasitärer Befall – in die differenzialdiagnostischen Überlegungen einbezogen werden sollten.

Somit lässt sich zusammenfassend sagen:

- Bei klinischem Verdacht auf Appendizitis und insbesondere bei fehlenden laborchemischen Entzündungszeichen sollte an einen Befall mit *Enterobius vermicularis* (*E. vermicularis*) gedacht werden.
- Eine reaktive Appendikopathie kann klinisch eine akute Appendizitis imitieren.
- Die Diagnose des Wurmbefalls erfolgt primär mittels perianalen Scotch-Tape-Tests; bildgebende Verfahren können ergänzend eingesetzt werden und unterstützen bei Verdacht auf eine reaktive Appendikopathie.
- Neben der postoperativen antiparasitären Behandlung und strikten Einhaltung der Hygienemaßnahmen bei dem Patienten ist eine Ausweitung dieser Maßnahmen auf alle engen Kontaktpersonen notwendig.
